# Magnesium implantation as a continuous hydrogen production generator for the treatment of myocardial infarction in rats

**DOI:** 10.1038/s41598-024-60609-2

**Published:** 2024-05-14

**Authors:** Bin Wang, Shuang Pan, Chaoqun Nie, Rentong Zou, Jiaren Liu, Xue Han, Li Dong, Jiawen Zhang, Xinrui Yang, Mengshu Yu, Bowei Fan, Xiaojian Hong, Wei Yang

**Affiliations:** 1 Fourth Affiliated Hospital of Harbin Medical University, Harbin, China; 2 Yuhuangding Hospital, Yantai, China; 3 Yantai Affiliated Hospital of Binzhou Medical University, Yantai, China

**Keywords:** Apoptosis, Hydrogen, Magnesium implantation, Myocardial infarction, Oxidative stress, Biomedical materials, Acute coronary syndromes, Heart failure, Vascular diseases, Implants

## Abstract

Molecular hydrogen is an emerging broad-spectrum antioxidant molecule that can be used to treat myocardial infarction (MI). However, with hydrogen inhalation, the concentration that can be reached within target organs is low and the duration of action is short, which makes it difficult to achieve high dose targeted delivery of hydrogen to the heart, seriously limiting the therapeutic potential of hydrogen for MI. As a result of reactions with the internal environment of the body, subcutaneous implantation of magnesium slices leads to continuous endogenous hydrogen production, leading to a higher hydrogen concentration and a longer duration of action in target organs. In this study, we propose magnesium implant-based hydrogen therapy for MI. After subcutaneous implantation of magnesium slices in the dorsum of rats, we measured hydrogen production and efficiency, and evaluated the safety of this approach. Compared with hydrogen inhalation, it significantly improved cardiac function in rats with MI. Magnesium implantation also cleared free radicals that were released as a result of mitochondrial dysfunction, as well as suppressing cardiomyocyte apoptosis.

## Introduction

Myocardial infarction (MI) is a severe form of cardiovascular disease that significantly impacts human health worldwide. Despite advances in the medical treatment of MI, innovative therapies are crucial to enhance the treatment outcomes of affected patients^1^.

Recently, hydrogen gas has emerged as a potential therapeutic agent for MI due to its anti-inflammatory and anti-oxidant properties, which can neutralize free radicals^2^. Currently, inhalation is the most commonly used administration route for hydrogen. However, the concentration of inhaled hydrogen is restricted to < 4% to prevent combustion, which consequently limits the attainment of higher hydrogen concentrations in target organs. Moreover, hydrogen easily diffuses in the body, meaning that its half-life is short, which necessitates continuous hydrogen intake and restricts the therapeutic efficacy of hydrogen inhalation. Consequently, there is an urgent demand for a novel approach to overcome these limitations.

Magnesium is a biocompatible material, and its alloys have achieved widespread use as implantable materials in clinical practice. Notably, magnesium possesses a unique ability to generate hydrogen gas as part of the following reaction upon contact with water in the body: Mg + 2H_2_O → Mg(OH)_2_ + H_2_. Therefore, magnesium functions as an in vivo hydrogen generator that continuously produces a high concentration of hydrogen gas^3^. Previous studies have demonstrated that subcutaneous implantation of magnesium creates a hydrogen-rich cavity, and due to the high diffusion ability of hydrogen, it can readily penetrate bodily tissues to reach the target site^4^.

In this study, we investigated the safety and efficacy of magnesium implant-based hydrogen therapy for MI in a rat model of MI. Our results suggest that magnesium implant-based hydrogen therapy is a safe and effective treatment option for MI. The approach mitigated free radicals generated from mitochondrial dysfunction, reduced the inflammatory response, and inhibited cardiomyocyte apoptosis. As a result, it improved cardiac function and reduced cardiac damage. Overall, our findings demonstrate that magnesium implantation is a more effective treatment for MI than traditional hydrogen inhalation therapy.

## Materials and methods

### Material preparation and sterilization

Magnesium slices were obtained from Dongguan Ruihe Ribiao Standard Metal Materials Co. Ltd., China. The high-purity (99.995%) magnesium ingots were processed into 2 × 2 × 1-mm slices using a combination of multiple-pass room temperature drawing, annealing techniques, and wire electrical discharge machining. To remove organic matter, the surface of the magnesium tablets was washed for 10 min with acetone, followed by a 10-min wash with anhydrous ethanol using an ultrasonic device. Prior to implantation, the magnesium slices were sterilized with 75% ethanol, and the surface of the magnesium slices was disinfected using ultraviolet light exposure for 30 min. The quality changes before and after magnesium slices implantation were measured using a high-precision electronic balance (Sensitivity 0.1 mg).

### Rat model of MI

Male Sprague–Dawley rats weighing 190–210 g were obtained from Beijing Vital River Laboratory Animal Technology Co. Ltd., Beijing, China. All experimental protocol were approved by the Institutional Animal Care and Use Committee of Harbin Medical University (IACUC-2021112). All animal procedures were performed in accordance with the relevant guidelines for the ethical treatment of animals. All animal methods were performed in accordance with ARRIVE guidelines. To induce MI, the rats were intubated and anesthetized with 1% pentobarbital solution (40 mg/kg), and buprenorphine (0.05 mg/kg) was administered intraperitoneally for perioperative analgesia. Left intercostal thoracotomy was performed to partially expose the heart, and the left anterior descending coronary artery was ligated using a 6-0 nylon suture to induce ischemia. The presence of MI was confirmed by immediate changes, including sudden pallor and paralysis of the affected part of the left ventricle, as well as ST-T segment elevation on electrocardiography. The sham (control) group underwent the same surgical procedure without ligation of the left anterior descending coronary artery. Penicillin sodium (80,000 U/rat) was administered intraperitoneally in all experiments to prevent postoperative infection. After 7 days, the rats were sampled.

### Implantation procedure of magnesium

After anesthesia, rats in the magnesium implant group were fixed on a heating pad. Following surgical guidelines, a 20 mm longitudinal incision was made along the midline of the dorsal skin. A subcutaneous pocket was created, and a magnesium slice (20 × 20 × 1 mm) was inserted. The pocket was rinsed with diluted penicillin solution before suturing. Rats in the sham surgery group underwent the same procedure without magnesium implantation.

### Echocardiographic evaluation of cardiac structure and function

The Philips high-resolution HD11 XE Ultrasound System with an S12-4 (4–12-MHz) probe (Philips Healthcare Ultrasound, the Netherlands) was used to perform echocardiography for cardiac function assessment. A cardiologist specializing in cardiac imaging conducted all echocardiographic evaluations. Left ventricular function was assessed in the parasternal short-axis view in the left lateral decubitus position using M-mode echocardiography. To examine left ventricular systolic function, ejection fraction (EF) and fractional shortening (FS) were measured. In addition, left ventricular internal diameter at end-diastole (LVIDd) and left ventricular internal diameter at end-systole (LVIDs) were measured to evaluate changes in cardiac structure. The average values of three successive cardiac cycles were used in the analysis. The subcutaneous structure of the gas cavity was also explored using this equipment.

### X-ray evaluation of the subcutaneous gas cavity

After administering anesthesia, the rats were positioned in the prone posture on a specialized pad, and digital subtraction angiography was performed (Artis zee ceiling, Siemens, Germany) using the advanced card3040 examination mode. The radiation head was precisely rotated to the tangent position of the magnesium tablet, enabling accurate recording of both the gas cavity and the image area with the magnesium tablet.

### Inhalation of 4% hydrogen

Rats in the 4% hydrogen inhalation group were administered 4% (v/v) hydrogen for 3 h once daily through a self-made device, hydrogen from a hydrogen generator and air from an air generator were regulated using flow meters and mixed in a plastic box. The concentration of hydrogen in the mixed gas was verified using a hydrogen detector (XP-3140, New Cosmos Electric Co. Ltd., Japan). Rats in the control, model, and magnesium implant groups were simultaneously administered air from an air generator through a similar device.

### Hydrogen concentration monitoring in the heart

The rats were anesthetized with 20% urethane (7 mL/kg) via intraperitoneal injection and supported with a ventilator throughout the hydrogen monitoring process. Real-time monitoring of the hydrogen concentration in the organs was performed using a Clark-type hydrogen microsensor (Unisense, Aarhus N, Denmark) equipped with a sensing anode (tip diameter: 50 μm). The tip of the microsensor was carefully inserted at a depth of 1 mm into the exposed heart, liver, and spleen. The negative current output of the hydrogen sensor was calibrated to determine the regional hydrogen concentration by referencing a calibration curve generated from hydrogen-saturated saltwater produced at a standard atmospheric pressure and a temperature of 25 °C.

### Transmission electron microscopy (TEM)

Fresh samples of left ventricular free wall myocardium (approximately 1 mm^3^) were immediately fixed in 2.5% glutaraldehyde at 4 °C overnight. Subsequently, ultrathin sections were obtained from the fixed blocks and processed according to standard TEM procedures.

### Scanning electron microscopy (SEM) and energy dispersive X-ray spectroscopy (EDS)

The surface morphology and chemical composition of the samples were characterized by SEM using the Hitachi SU5000 equipped with EDS. The 20 × 20 × 2-mm rectangular magnesium samples, which had been implanted subcutaneously, were first polished with 1200 grit silicon carbide paper and then ultrasonically cleaned in acetone, ethanol, and deionized water for 10 min each to remove surface impurities and organic residue. After retrieval, the samples were rinsed with acetone, ethanol, and deionized water again before being characterized using the SEM–EDS system.

### 2,3,5-Triphenyltetrazolium chloride (TTC) staining and infarct size measurement

After anesthesia, the MI group with hydrogen inhalation (MI + hydrogen group) and the MI group with magnesium implantation (MI + magnesium group) underwent chest opening. The hearts were rapidly frozen at − 20 °C for 15 min and then sectioned into five-axis slices (2 mm) from the apex to the bottom. The sections were then incubated at 37 °C in 2% TTC (Solarbio, Beijing, China) for 20 min. The viable myocardial tissue was visually distinguishable in red, while the non-viable myocardial tissue appeared white. The cardiac sections were then washed three times with phosphate-buffered saline (PBS) and imaged using a digital camera. Subsequently, the total left ventricular area and the infarct area were quantified using Image J software, version 8.4 (National Institutes of Health, US).

### Evaluation of the pressure and area of the gas cavity

A manual tonometer was used to evaluate the pressure of the gas cavity. The highest point within the gas cavity was standardized as the focal point for examination. The X-ray film of the magnesium tablet in the long-axis view was selected for analysis. The area of the gas cavity was assessed using Image J software. All recorded data were analyzed for at least three rats.

### Histopathology, immunofluorescence, reactive oxygen species (ROS) detection, and TUNEL staining

The fixed cardiac tissue samples were stained with hematoxylin and eosin (H&E). Immunofluorescence staining was used to explore 8-hydroxy-2′-deoxyguanosine (8-OHdG) expression in the cardiac tissue samples. After treatment, the samples were inoculated overnight with rabbit anti-8-OHdG antibody (1:1000, Bioss, bs-1278R, Beijing, China). For ROS detection, BBoxiProbe® DHE fluorescent probe for ROS was diluted 1000-fold with distilled water, and 10-μm-thick frozen sections were treated with 200 μL washing solution, followed by removal of the washing solution and addition of 200 μL staining solution. The sections were incubated at 37 °C in the dark for 30 min before washing with PBS. TUNEL staining (Roche, US) was performed to determine apoptosis in the cardiac tissues according to the manufacturer’s instructions. Briefly, frozen tissue sections were fixed with 4% paraformaldehyde, permeabilized with 0.1% Triton X-100, and subjected to the TUNEL reaction mixture for 1 h at 37 °C. All stained sections were imaged by fluorescence microscopy (Olympus, Tokyo, Japan). Three fields were randomly selected for observation. All images were examined in a blinded manner.

### Malondialdehyde (MDA)

Malondialdehyde (MDA) is a product of lipid peroxidation that is commonly used to assess the degree of cellular and tissue oxidative stress. We measured MDA levels in the cardiac tissues of rats to evaluate the extent of oxidative stress. MDA was measured using a commercial assay kit (BC0025, Solarbio) following the manufacturer’s instructions.

### Enzyme-linked immunosorbent assay (ELISA)

After the collection of cardiac and peripheral blood samples, the blood serum and red blood cells were rapidly and carefully separated by centrifugation at 1000×*g* for 10 min. The concentrations of tumor necrosis factor (TNF)-α (Abbkine, Wuhan, China), interleukin (IL)-1β (Abbkine), IL-6 (Abbkine), cardiac troponin I (cTnI) (Nanjing Jiancheng BioEngineering Institute, Nanjing, China), and brain natriuretic peptide (BNP) (Nanjing Jiancheng BioEngineering Institute) in the peripheral blood were determined using commercially available ELISA kits according to the manufacturer’s instructions.

### Biochemical and electrolyte tests

Blood urea nitrogen (BUN) and uric acid (UA) biochemical tests were performed, and sodium, chloride, potassium, magnesium, and calcium were measured using the Fully Automated Biochemical Analysis System (ARCHITECT c16000, Abbott, US).

### Determination of the mitochondrial membrane potential (MMP)

The mitochondria were extracted from cardiac tissues using the mitochondrial extraction kit (SM0020, Solarbio). The MMP assay was performed following the manufacturer’s instructions. The MMP was determined using 5 µM JC-1 (Beyotime, Shanghai, China). After rinsing with JC-1 washing buffer, fluorescence was measured using a microscope (Olympus).

### Western blot analysis

Total protein was extracted using RIPA buffer (Beyotime). Samples containing at least 5 μg protein from each group were separated by sodium dodecyl sulfate–polyacrylamide gel electrophoresis and transferred to polyvinylidene fluoride (PVDF) membranes. After blocking, the PVDF membranes incubated with various primary antibodies at 4 °C overnight, including antibodies against Bax (1:1000, ab182733, Abcam, US), Bcl-2 (1:1000, ab182858, Abcam), cleaved caspase-3 (1:1000, 19677-1-AP, Proteintech, China), cleaved caspase-9 (1:1000, ab184786, Abcam), cytochrome c (1:1000, ab133504, Abcam), and GAPDH (1:4000, AB2000, Abways, China). The membranes were incubated with the corresponding conjugated secondary antibodies (1:2000, ZSGB-BIO, ZB-2301, Beijing, China). Membranes were cut because target protein with similar molecular weights required eluting and re-incubating. Then, the membranes were visualized by enhanced chemiluminescence (Vazyme, Nanjing, China). The protein was quantified using Image Lab software 6.1 (Bio-Rad Laboratories Co., Ltd. US).

### Statistical analysis

Data were analyzed for normal distribution by the Shapiro–Wilk test. Two datasets meeting the normal distribution and equal variances warrant the use of an independent samples t-test for comparison. Differences among the groups were assessed using one-way analysis of variance followed by least significant difference post-hoc test. Continuous parameters were normally distributed and data were presented as mean ± standard deviation (SD).P value of < 0.05 was considered statistically significant. All data were analyzed using GraphPad Prism 9.0.0 for Windows (GraphPad Software Inc., US) (Supplementary Information).

## Results

### Magnesium degradation and gas cavity formation after implantation

**Figure 1:**
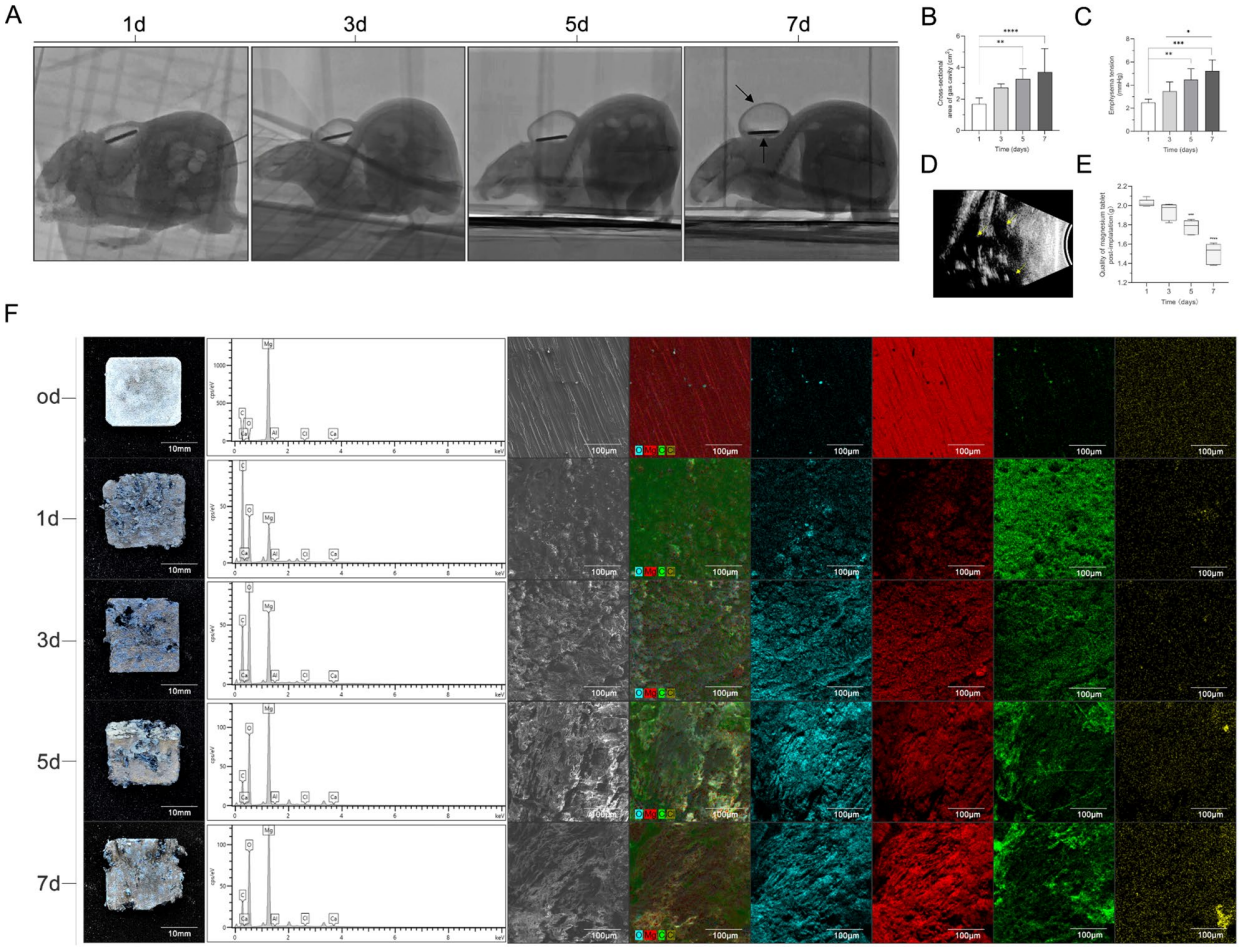
Magnesium degrades and forms gas cavities after magnesium slice implantation in rats. X-ray image of the gas cavity changes over time after magnesium slice implantation. The X-ray central line is projected parallel to the long-axis of the magnesium slice. The time points were 1, 3, 5, and 7 days (**A**). The tension changes in the gas cavity were recorded at the same time points, and the cross-sectional area of the gas cavity was measured (n = 5) (**B**,**C**). Ultrasound examination of the gas cavity at 7 days. The yellow arrows pointing to the black area represent the subcutaneous multi-chamber gas cavity structure (**D**). Changes in the quality of the magnesium slices after implantation (**E**). Photographs; SEM images; EDS spectra; and elemental mapping of oxygen, magnesium, carbon, chloride, and calcium were used to investigate magnesium slices corrosion before (0 days) and after implantation. The magnesium slices were systematically retrieved and thoroughly cleaned at specific time points (1, 3, 5, and 7 days) after implantation (**F**). EDS, energy-dispersive X-ray spectroscopy; SEM, scanning electron microscopy. The data are presented as the mean ± standard deviation.*p < 0.05, **p < 0.01, ***p < 0.001, and ****p < 0.0001.

Each rat underwent aseptic surgery for subcutaneous implantation of a magnesium slice (2000 mg). Magnesium with a purity exceeding 99.9% was chosen to avoid any unknown effects of other additive elements on the body. Visible gas cavities appeared 1 day after magnesium implantation (Fig. 1A), and morphological enlargement of the gas cavities was observed on days 3, 5, and 7. X-ray transmission imaging showed an increasing amount of gas (Fig. 1B). Simultaneously, the surface tension of the gas cavities gradually increased (Fig. 1C). Both the tension and the area of the gas cavities exhibited a significant increase on days 5 and 7. Gas permeation and the structure of the subcutaneous multi-chamber gas cavities were observed in the tissue interstice surrounding the implant (Fig. 1D). To investigate the degradation of the magnesium implant in vivo, we retrieved the implanted magnesium slices from the rats at four time points (1, 3, 5, and 7 days) and cleaned the slices with acetone and alcohol. The degradation rate of the implanted magnesium slices in vivo increased significantly from days 5–7 (Fig. 1E). SEM–EDS provided information on the surface morphology and chemical composition of the implanted magnesium slices and their interaction with the surrounding tissues (Fig. 1F). The EDS analysis of the implanted magnesium slices revealed the presence of magnesium, carbon, oxygen, and chloride. The magnesium peak was the most prominent in both the implanted and non-implanted slices, indicating the purity of the magnesium slices used. The presence of carbon and oxygen in both the implanted and non-implanted magnesium slices could be attributed to surface contamination or oxidation products resulting from exposure to the environment. The presence of chloride may suggest the formation of calcium chloride, which is a common by-product of inflammation. Additionally, the detection of chloride indicated the occurrence of localized corrosion, which was supported by the formation of corrosion pits on the surface of the magnesium slices.

### Diffusion of hydrogen gas from the gas cavity into the organs

**Figure 2:**
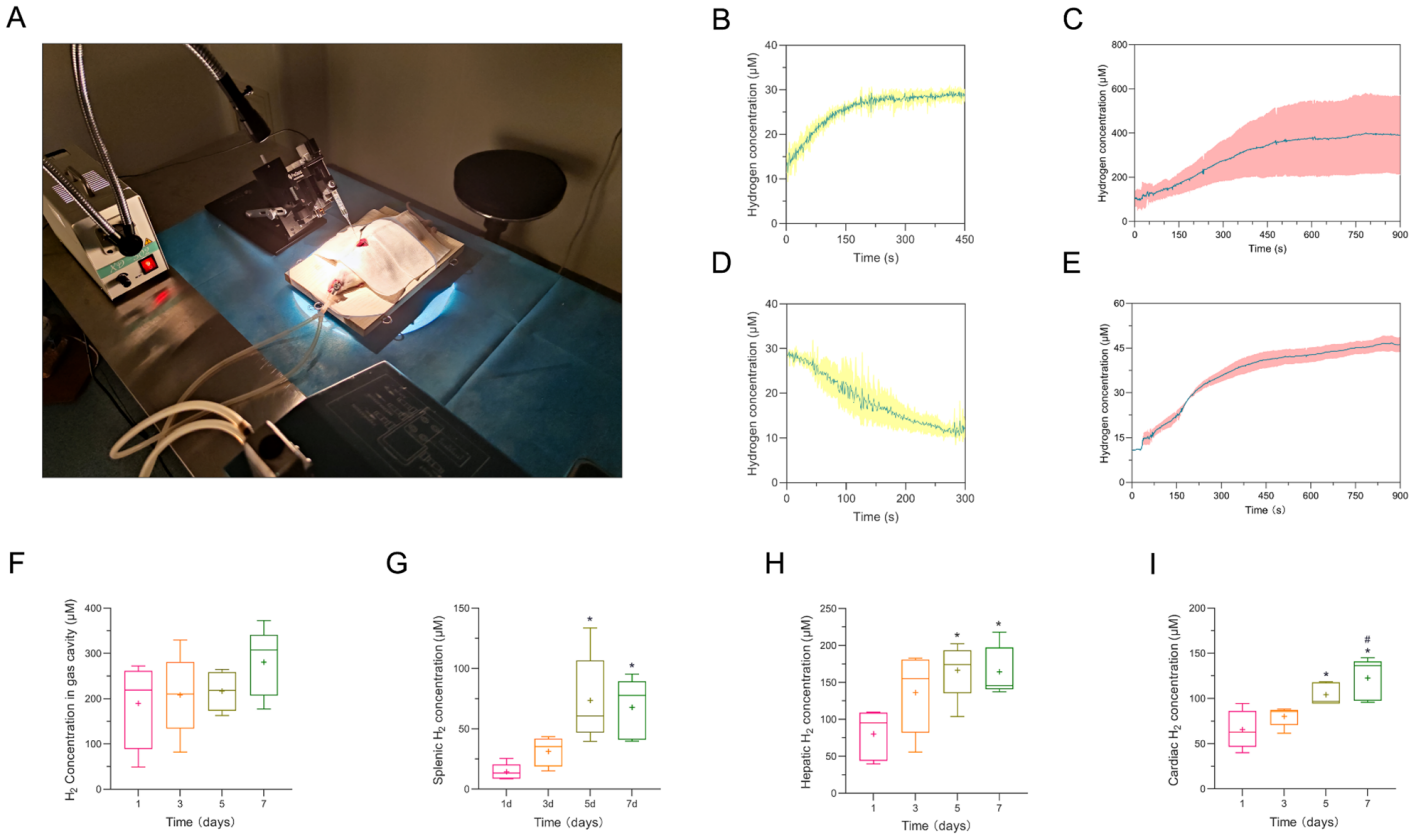
Hydrogen gas within the cavities diffuses into the organs of rats. High-resolution photographs were taken to document the process of detecting the hydrogen concentration in the organs of anesthetized rats undergoing surgery with ventilatory support (**A**). Hydrogen concentration in the hearts of rats during inhalation of 4% hydrogen (n = 3), specifically at the initiation of hydrogen inhalation (**B**) and after its cessation (**D**). The hydrogen concentrations in the gas cavity (**C**) and heart (**E**) were assessed after magnesium slice implantation (n = 3). The concentrations of hydrogen in the gas cavity and tissues of the liver, heart, and spleen at 1, 3, 5, and 7 days after magnesium slice implantation (**F–I**). The data are presented as the mean ± standard deviation.*p < 0.05, **p < 0.01, ***p < 0.001, and ****p < 0.0001.

We exposed the organs through sterile surgical procedures, allowing direct measurement of the hydrogen concentration using hydrogen electrodes (Fig. 2A). The method used for hydrogen inhalation differed from that used for magnesium slice implantation. Hydrogen is a highly permeable gas molecule, but it cannot be stored in the body for an extended period. Upon inhaling 4% hydrogen, the concentration of hydrogen in the heart rapidly increased, reaching a plateau at 30 μmol/L within approximately 5 min (Fig. 2B). After cessation of hydrogen inhalation, the concentration of hydrogen in the heart sharply decreased to baseline within 5 min (Fig. 2D). Surprisingly, the hydrogen concentration in the heart could be detected immediately after magnesium slice implantation, slowly reaching a plateau 15 min later. Although the increase in the hydrogen concentration occurred more slowly than with hydrogen inhalation, the hydrogen concentration in the heart after magnesium slice implantation reached 45 μmol/L, which was higher than the maximum hydrogen concentration in the heart of 30 μmol/L achieved with hydrogen inhalation (Fig. 2C). We measured the hydrogen concentration in the gas cavity after magnesium slice implantation, recording only the data at the highest point of the gas cavity. The hydrogen concentration curve was similar to that of the hydrogen concentration in the heart, showing a slow increase and reaching a plateau at around 15 min (Fig. 2E). To explore hydrogen production and the stability of the hydrogen concentration in the organs after magnesium slice implantation, we measured the hydrogen concentration in the heart, liver, spleen, and gas cavity at 1, 3, 5, and 7 days after magnesium implantation (Fig. 2F–I). The hydrogen concentration in the heart, liver, and spleen showed an increasing trend over time, and all exhibited a significant increase in hydrogen concentration at 5 and 7 days.

### Biocompatibility of magnesium slice implantation

**Figure 3:**
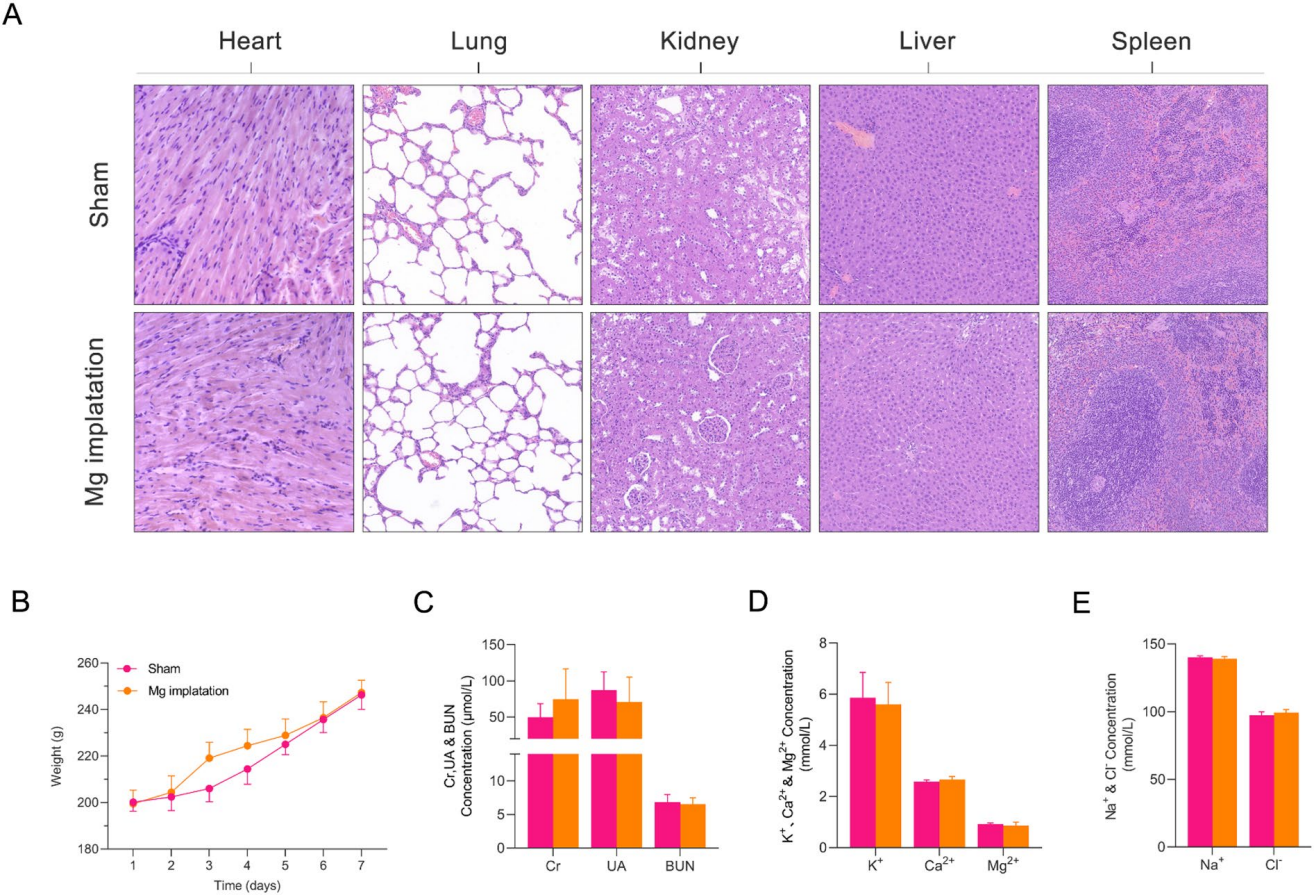
Histological findings and electrolyte maintenance after magnesium slice implantation. H&E staining of the heart, lungs, kidneys, liver, and spleen harvested from rats in the sham and magnesium implantation groups at 7 days after MI (**A**). Weight changes in rats within 7 days after magnesium slice implantation (**B**). Changes in serum Cr, BUN, UA, K^+^, Ca^2+^, Mg^2+^, Na^+^, and Cl^−^ 7 days after magnesium slice implantation (**C–E**). The data are presented as the mean ± standard deviation, n = 5, blood urea nitrogen; *Ca*^*2*+^ calcium, *Cl*^*−*^ chloride, *Cr* creatinine, *H&E* hematoxylin and eosin, *K*^+^ potassium, *Mg*^*2*+^ magnesium, *Na*^+^ sodium, *UA* uric acid. The data are presented as the mean ± standard deviation and were analyzed by one-way analysis of variance.

To investigate the safety and feasibility of magnesium slice implantation, the rats were divided into two groups. In the magnesium implantation group, magnesium slices were implanted subcutaneously on the dorsum of the rats. In the sham group, rats underwent a superficial incision on the back without implanting any magnesium slices. We performed H&E staining of relevant organs involved in magnesium metabolism, including the heart, lungs, kidneys, liver, and spleen. We also measured changes in serum electrolytes and metabolic indicators of organ function to explore the effects of magnesium implantation on the histology and serum biochemistry of rats. H&E staining of the heart, liver, spleen, kidney, and lungs from the magnesium implantation group and the sham group showed no significant pathological differences. Specifically, the liver tissue displayed regular hepatic lobule structures with hepatocytes of uniform size and shape, nuclei that were round or oval in shape, and cytoplasm that contained scattered small vacuoles or oil droplets. The spleen consisted of red and white pulp regions with uniformly sized/shaped splenic corpuscles, and no abnormal structures or inflammatory reactions were observed. The renal tubular epithelium displayed a tightly packed brush border and longitudinal stripes at the base of the cells. The glomerulus exhibited a well-defined spherical structure, with a diameter of approximately 100 μm, which was enveloped by a capsule containing a capillary network. Intact red blood cells were observed passing through the glomerulus, and no signs of inflammatory cell infiltration or fibrosis formation were observed. The lung tissue displayed regular alveolar structures with thin and orderly alveolar walls, and no alveolar collapse, congestion, or edema was observed (Fig. 3A). Within 1 week, both groups of rats showed no significant abnormalities in diet, activity, or sleep. The curves of body weight changes were similar between the groups (Fig. 3B). We compared the serum concentrations of potassium, sodium, chloride, calcium, magnesium, BUN, creatinine, and UA between the magnesium implantation group and the sham group. No significant differences in these parameters were identified. However, we did observe that the serum magnesium concentration in the magnesium implantion group did not increase compared with the sham group (Fig. 3C–E).

### Magnesium implantation reduces MI and improves cardiac function

**Figure 4:**
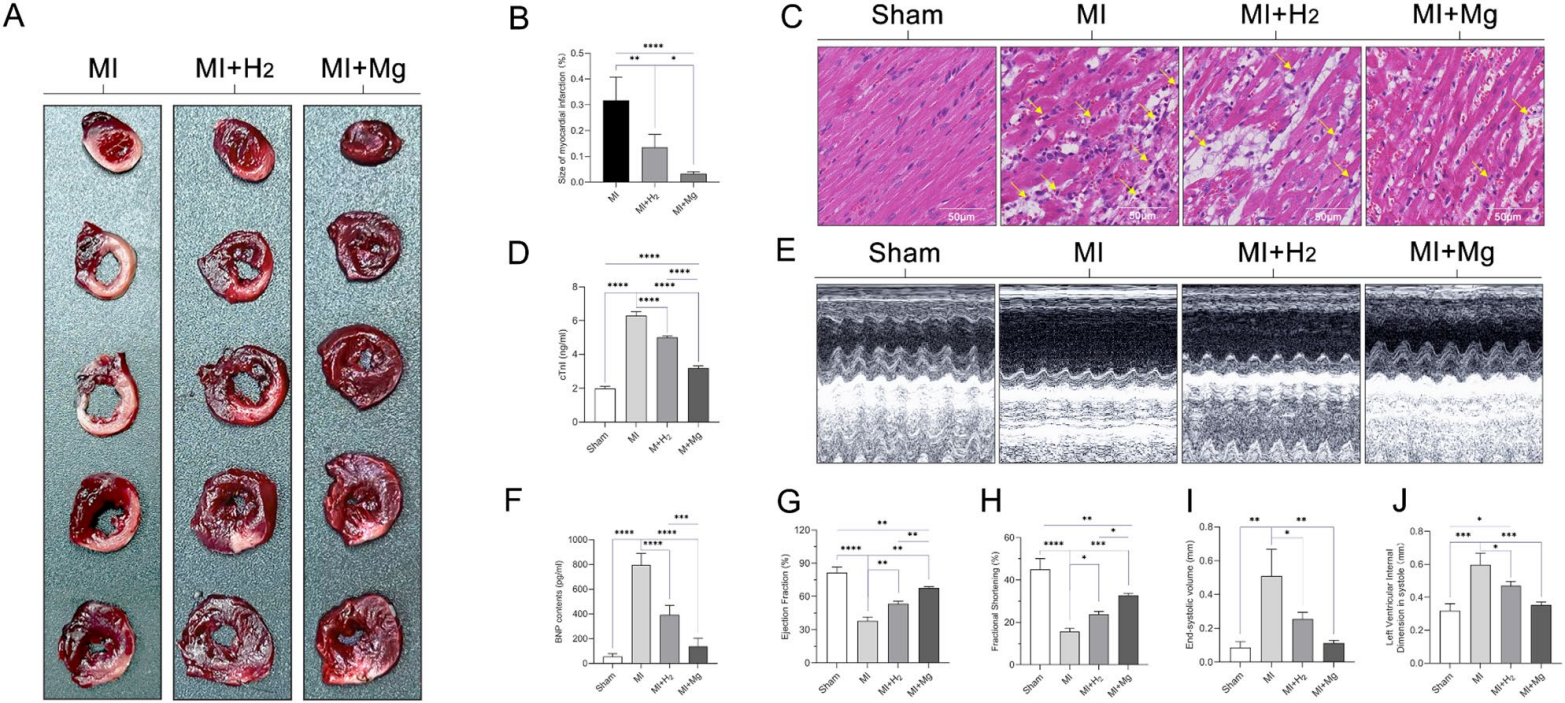
Effects of magnesium slice implantation on cardiac function and myocardial structure in rats. Representative TTC staining images and quantitative analysis in each group (**A,B**). Representative H&E staining images in each group. Scale bar: 50 µm. The arrows indicate inflammatory cell infiltration (**C**). Serum concentration of the cardiac injury marker cTnI (**D**). Representative M-mode echocardiograms obtained from the sham, MI, MI + hydrogen, and MI + magnesium groups (**E**). The concentration of BNP as a serum biomarker of heart failure (**F**). Summary data for echocardiographic measurements in each group of animals, including EF (**G**), LVIDs (**H**), ESV (**I**), and FS (**J**). The data are expressed as the mean ± standard deviation (n = 5 rats per group). *p < 0.05, **p < 0.01, ***p < 0.001, and ****p < 0.0001. *BNP* brain natriuretic peptide, *cTnI* cardiac troponin I, *EF* ejection fraction, *ESV* end-systolic volume, *FS* fractional shortening, *LVIDs* left ventricular internal diameter at end-systole, *H&E* hematoxylin and eosin, *MI* myocardial infarction, *TTC* 2,3,5-triphenyltetrazolium chloride.

We used TTC staining to assess infarct size in three groups of rats: MI, MI + hydrogen, and MI + magnesium. We ensured comparability of infarct size between the groups by cutting the cardiac tissues into five levels at equal distances. Both the MI + hydrogen and MI + magnesium groups demonstrated significantly smaller infarct sizes than the MI alone group 7 days after MI. The MI + magnesium group (0.03321 ± 0.007746) exhibited a significantly more pronounced reduction in infarct size than the MI + hydrogen group (0.1351 ± 0.04981) (P < 0.05) (Fig. 4A,B). Histopathologic examination of the rat hearts obtained 7 days after MI showed varied degrees of necrosis and disorientation, and swelling in the cardiac muscle fibers, edema, and marked inflammatory cell infiltration were also observed compared with the sham group. The MI alone group exhibited the most pronounced inflammatory cell infiltration, followed by the MI + hydrogen group, while the MI + magnesium group showed the least inflammatory cell infiltration (Fig. 4C). cTnI is considered one of the important biomarkers of myocardial injury. Rats with MI showed a significant increase (P < 0.001) in cTnI compared with the sham group. Both the MI + hydrogen and the MI + magnesium groups demonstrated a significant decrease (P < 0.001) in cTnI compared with the MI alone group. The effect of reducing cTnI in magnesium slice implantation was superior to that of hydrogen inhalation (Fig. 4D). We assessed systolic function in all groups at 7 days after MI using two-dimensional and M-mode echocardiography (Fig. 4E). Both hydrogen inhalation and magnesium slice implantation resulted in a significant improvement in left ventricular systolic function. The diagnostic outcomes of the heart failure biomarker BNP aligned with the findings of echocardiography (Fig. 4F). The summary data for EF (Fig. 4G), FS (Fig. 4H), end-systolic volume (Fig. 4I), and LVIDs (Fig. 4J) demonstrated significant improvements in the MI + hydrogen group and the MI + magnesium group at 7 days after MI compared to the MI alone group. Furthermore, the MI + magnesium group exhibited superior effects over the MI + hydrogen group in terms of EF and FS. These findings suggest that magnesium implantation has a superior effect on cardiac function following MI when compared with hydrogen inhalation.

### Improvement in mitochondrial injury, inflammation, and oxidative stress in rats with MI after magnesium slice implantation

**Figure 5:**
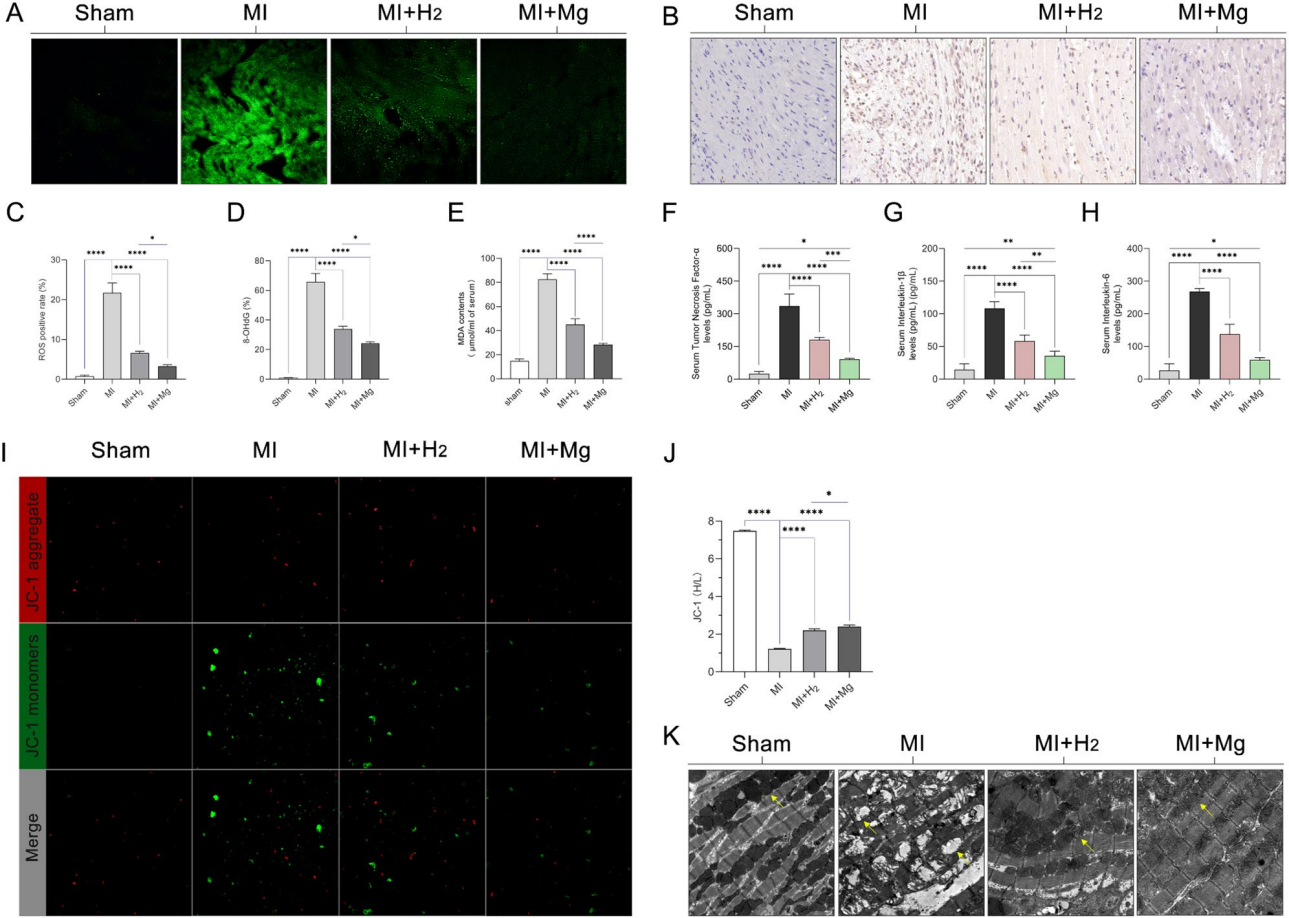
The effect of magnesium implantation on cardiac oxidative stress, inflammation, and mitochondrial damage in rats with MI. Representative photomicrographs of rat myocardial tissue stained with green fluorescent probe for total ROS semi-quantification and statistical analysis presented in graphical form. Bar: 50 µm (**A,C**). Representative 8-OHdG staining images and quantitative analysis in each group (**B,D**). Myocardial tissue MDA content in rats (**E**). Quantification of cytokines in rat myocardial tissue (TNF-α, IL-1β, and IL-6) (**F–H**). Changes in MMP in purified mitochondria stained with JC-1. Red and green fluorescence represent the aggregated and monomeric forms of JC-1, respectively (**I**). The ratio of red to green fluorescence intensity was calculated (**J**). Electron microscopy images of rat cardiac tissue. The yellow arrows point to the mitochondria. Magnification: × 2000 (**K**). The data are presented as the mean ± standard deviation. The Student’s t-test was used for comparisons between groups, n = 5, *p < 0.05, **p < 0.01, ***p < 0.001, and ****p < 0.0001. *IL* interleukin, *MDA* malondialdehyde, *MMP* mitochondrial membrane potential, *MI* myocardial infarction, *ROS* reactive oxygen species, *TNF* tumor necrosis factor, *8-OHdG* 8-hydroxy-2′-deoxyguanosine.

We examined the effect of magnesium implantation on total ROS levels in rats with MI using frozen sections stained with a ROS-sensitive probe (Fig. 5A). ROS levels were quantified using Image J software after background subtraction. Compared with the sham group, the MI group demonstrated a significant increase in ROS fluorescence, while both the MI + hydrogen group and the MI + magnesium group showed a significant reduction in ROS fluorescence. Notably, the MI + magnesium group exhibited a more pronounced reduction in ROS (Fig. 5C). Changes in intracellular nucleic acid oxidative stress levels were assessed through immunohistochemical staining of 8-OHgG (Fig. 5B). In cardiomyocytes from the MI group, positive staining for 8-OHdG was significantly increased compared with the sham group, indicating damage caused by oxidative stress. Positive staining for 8-OHdG was weaker in cardiomyocytes from the MI + hydrogen group and the MI + magnesium group, suggesting that oxidative stress was alleviated to some extent with hydrogen inhalation and magnesium slice implantation. Notably, in cardiomyocytes from the MI + magnesium group, positive staining for 8-OHdG was significantly reduced, indicating that magnesium implantation had a stronger inhibitory effect on oxidative stress (Fig. 5D). The MDA concentration was also quantified to assess alterations in lipid peroxidation within the serum on postoperative day 7 (Fig. 5E). Similar to the results of 8-OHdG staining and total ROS levels, the MI + magnesium group demonstrated a stronger anti-lipid peroxidation effect (P < 0.001). Furthermore, the extent of inflammation in each group was evaluated by quantifying inflammatory factors, including TNF-α, IL-1β, and IL-6, in the serum (Fig. 5F–H). The MI group showed markedly elevated inflammatory factor concentrations. However, following hydrogen inhalation and magnesium implantation, the concentrations of inflammatory factors decreased. Notably, the MI + magnesium group had significantly lower concentrations of TNF-α and IL-1β than the MI + hydrogen group (P < 0.001 and P < 0.01, respectively), suggesting that magnesium implantation exhibited a more potent anti-inflammatory effect. We simultaneously measured changes in the MMP in myocardial tissues using the JC-1 probe. When the MMP was normal, JC-1 existed in an aggregated state (red fluorescence), and when the MMP was decreased, JC-1 monomorphism increased, showing green fluorescence (Fig. 5I). In the MI group, the fluorescence intensity of JC-1 decreased significantly, indicating that the MMP decreased. In the MI + hydrogen group, the JC-1 fluorescence intensity was relatively stable, The change in MMP in the MI + magnesium group was more obvious (P < 0.05) than that in the MI + hydrogen group (Fig. 5J). Further ultrastructural analysis of cardiomyocytes was performed using electron microscopy in all groups. The MI group exhibited severe mitochondrial swelling and disorganized muscle fibers. In contrast, both the MI + hydrogen group and the MI + magnesium group showed significant improvements in mitochondrial morphology and partial recovery of muscle fiber structure. Specifically, the MI + magnesium group demonstrated a greater improvement than the MI + hydrogen group, with reduced mitochondrial swelling, morphology approaching a normal state, and more extensive recovery of disorganized muscle fibers (Fig. 5K). These findings suggest that magnesium implantation ameliorates oxidative stress, inflammation, and mitochondrial damage in acute MI in rats.

### Magnesium implantation reduces apoptosis in rats with MI

**Figure 6:**
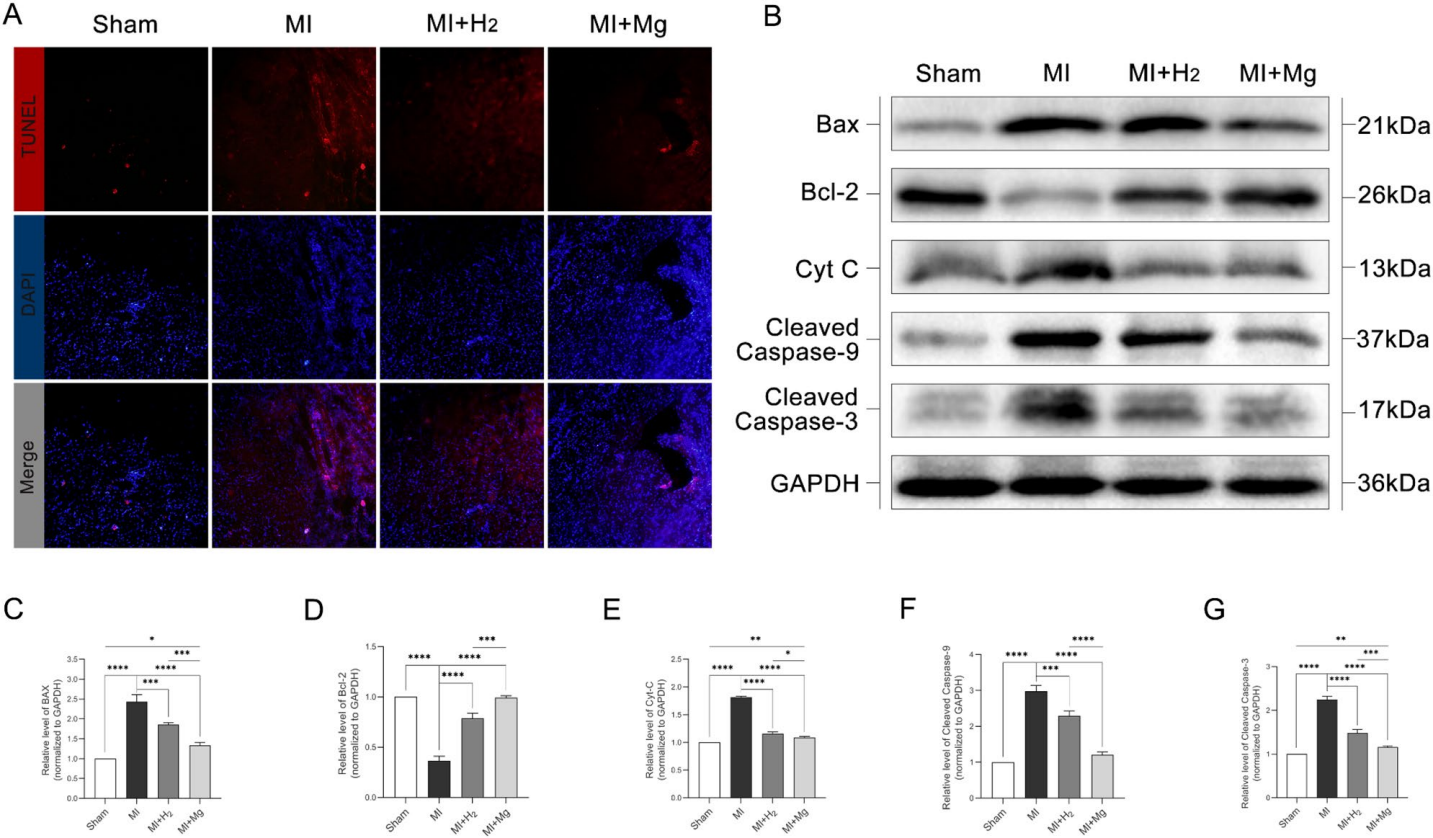
The impact of magnesium slice implantation on cardiomyocyte apoptosis in rats with MI. Cardiomyocyte death was evaluated by double staining in each group. Apoptotic cells (TUNEL-positive cells) are stained in red with TUNEL, and cardiomyocyte nuclei are labeled in blue with DAPI (**A**). Western blot analysis was performed to measure the expression of cleaved caspase-9, cleaved caspase-3, Bcl-2, Bax, and cytochrome c. GAPDH was used as the loading control (**B**). The densitometry results of cleaved caspase 9, cleaved caspase 3, Bcl-2, Bax, and cytochrome c/GAPDH are presented as the mean ± standard deviation of a minimum of three independent experiments. The data were analyzed by one-way analysis of variance (**C–G**). *p < 0.05, **p < 0.01, ***p < 0.001, and ****p < 0.0001, n = 3, *MI* myocardial infarction.

**Figure 7:**
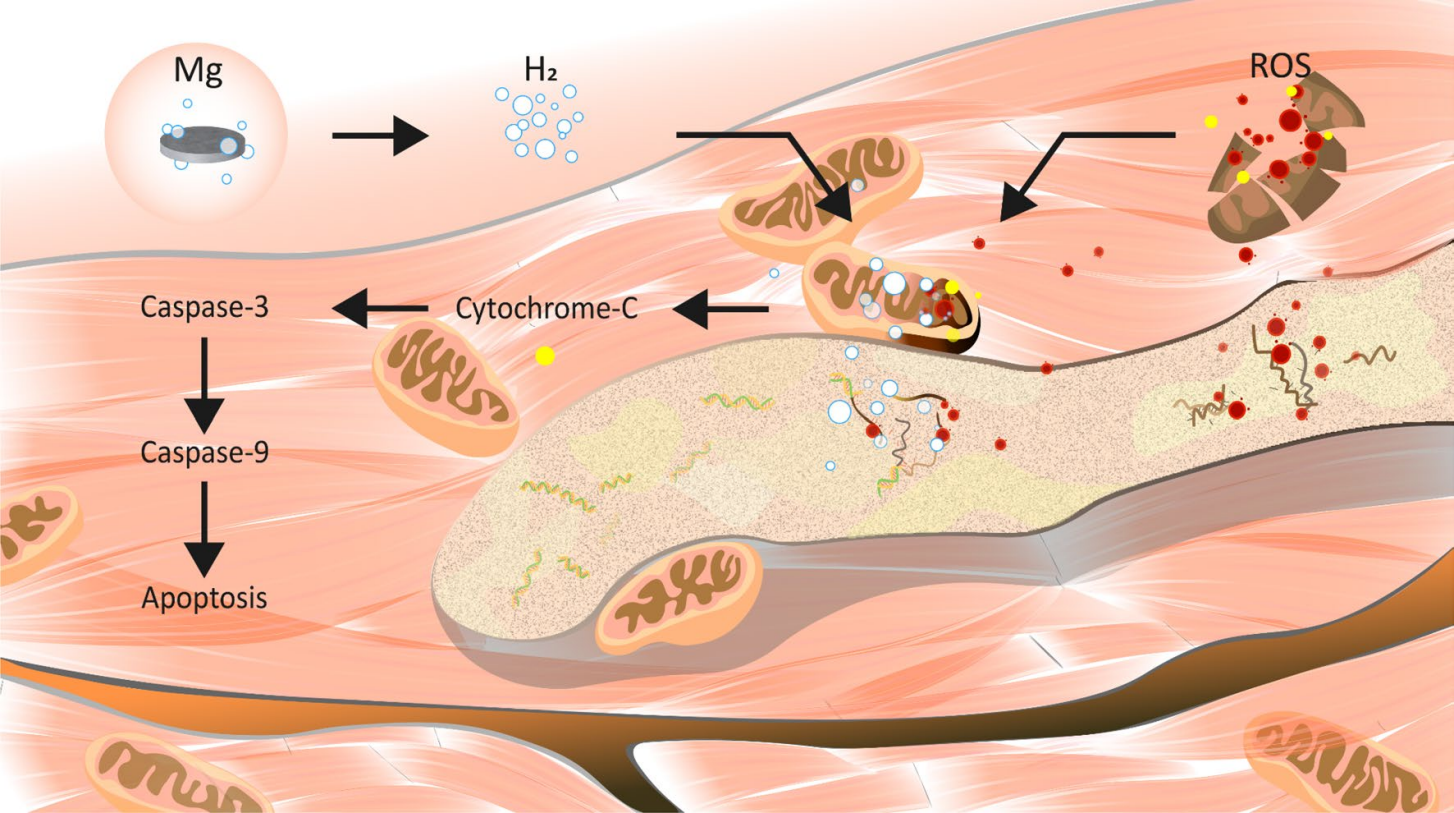
Schematic representation of hydrogen production resulting from magnesium slice implantation and its effects on the mitochondrial apoptosis pathway. Hydrogen production resulting from magnesium slice implantation improves cardiac oxidative stress and apoptosis after MI in rats. *MI* myocardial infarction.

The TUNEL assay showed an increase in the number of TUNEL-positive cells in myocardial tissues from rats with MI compared with rats in the sham group (Fig. 6A), indicating that MI induces cardiomyocyte apoptosis. The number of TUNEL-positive cells was lower in both the MI + hydrogen and MI + magnesium groups than in the MI alone group, suggesting that both interventions reduced cardiomyocyte apoptosis. Of note, magnesium implantation was more effective in reducing the number of TUNEL-positive cells than hydrogen inhalation, indicating that magnesium implantation was more effective in reducing cardiomyocyte apoptosis. Simultaneously, we measured the expression of apoptosis-related proteins, including Bax, Bcl-2, cytochrome c, cleaved caspase-3, and cleaved caspase-9, in all groups by Western blot analysis (Fig. 6B). The results show that the expression of Bax and cytochrome c was significantly higher in the MI group than in the sham group (Fig. 6C), indicating activation of the intrinsic apoptotic pathway. In contrast, Bcl-2 expression was downregulated (Fig. 6D). Treatment with hydrogen inhalation or magnesium implantation partially restored the balance between pro-apoptotic and anti-apoptotic proteins, as evidenced by the decreased expression of Bax and cytochrome c (Fig. 6E) and the increased expression of Bcl-2. In addition, the activation of caspase 3 and caspase 9 was also inhibited in the MI + hydrogen and MI + magnesium groups, indicating that the apoptotic cascade was inhibited (Fig. 6F–G). These results suggest that MI induces cardiomyocyte apoptosis, and that both hydrogen inhalation and magnesium implantation reduce cardiomyocyte apoptosis, with magnesium implantation exerting a more pronounced effect. Diagram illustrating the therapeutic effects of magnesium implantation in generating hydrogen molecules for treating myocardial infarction in rats (Fig. 7).

## Discussion

Hydrogen has been proven to have therapeutic effects in MI^5,6^. For patients with acute MI, hydrogen can be safely and effectively used to prevent adverse left ventricular remodeling^7^. Molecular hydrogen selectively neutralizes toxic free radicals without interfering with physiological ROS^2,8^. The therapeutic effects of hydrogen show a concentration–response relationship^9^, but due to safety concerns when the hydrogen concentration exceeds 5% in terms of the risk of combustion, the concentration of inhalational hydrogen is currently limited to 2–4%, restricting its therapeutic potential. To overcome this limitation, studies have explored new methods of hydrogen administration, such as using nanocarriers to deliver a large amount of hydrogen to the body^10^ or loading hydrogen inside microbubbles and rapidly releasing it at the cardiac location under ultrasound guidance^11^. These approaches increase the hydrogen concentration in target organs and demonstrate better effects compared with simple hydrogen inhalation. However, these methods are complex and costly, and hydrogen still has a short duration of action and requires repeat injection. In this study, we present a novel approach for endogenous hydrogen production via in vivo magnesium implantation to overcome the limitations of existing methods.

Based on the chemical equation Mg + 2H_2_O → Mg(OH)_2_ + H_2_, Mg(OH)_2_ is unstable in the high chloride environment of the body because it reacts with chloride ions to form a soft magnesium chloride film. Additionally, it reacts with carbonate and phosphate in the body and attaches to organic substances, making the in vivo reaction more complex than the reaction of magnesium with simulated body fluids, such as Ringer’s solution. Typically, the reaction rate of magnesium implantation in the body increases during the first week, peaks at 5–7 days, and then gradually decreases^12^. The present study showed that after magnesium implantation, there was visible enlargement of the gas cavity over time on X-ray imaging, with the gas cavity area and tension of the magnesium slice significantly increasing after 5 days. Subcutaneously, multiple gas-filled cavities were observed. We selected 1 week as the endpoint of the study to avoid potential interference with the organ hydrogen concentration caused by gas extraction from excessively enlarged cavities. This is important as previous research has shown that allowing emphysema to enlarge can increase animal mortality^13^. Within 1 week, the animals did not exhibit abnormal changes in body weight, indicating that their appetite and vitality were not significantly affected in the present study. The magnesium slices retrieved at fixed time points showed varying degrees of corrosion. At the endpoint (7 days), the mass of the magnesium slices had decreased by one fourth (from 2.025 ± 0.03989 g to 1.502 ± 0.1086 g), indicating that approximately 500 mL pure hydrogen gas was generated.

Hydrogen is a potential anti-oxidant exhibiting excellent distribution properties and having the physical ability to penetrate biological membranes and diffuse into the cytoplasm^14^. Hydrogen rapidly diffuses into the cytoplasm, cell nucleus, and mitochondria. Moreover, hydrogen can pass through the blood–brain barrier^15^. This is a feature that surpasses most anti-oxidant compounds. However, the high diffusion ability of hydrogen gas is a double-edged sword, meaning that hydrogen gas cannot stay in the target organ for a long period of time^6^. A previous study showed that the hydrogen concentration in rat blood and tissues reached its peak 30 min after hydrogen gas inhalation, and then gradually decreased between 30 and 60 min^16^. In this study, we compared hydrogen inhalation with hydrogen production resulting from magnesium implantation. We adopted the commonly used method of 4% hydrogen gas inhalation for 3 h daily^17^. We found that both inhaled hydrogen gas and hydrogen produced by magnesium implantation easily circulated within organs and was detected by the hydrogen electrode. After inhaling 4% hydrogen gas, the hydrogen concentration in the heart reached saturation within 5 min. At a normal temperature and pressure, the maximum saturation concentration of 4% hydrogen gas in the 0.9% sodium chloride fluid environment of the body is approximately 30 μmol/L. When hydrogen intake was terminated, the hydrogen concentration in the heart returned to baseline within 5 min, indicating that hydrogen gas rapidly escapes from the tissues^18^. After magnesium slice implantation, hydrogen gas can also be rapidly generated and detected immediately in the skin and organs, as shown previously^4^. The subcutaneously implanted magnesium slice forms a high-concentration hydrogen gas zone within 15 min. The hydrogen gas rapidly diffuses along the concentration gradient to surrounding organs. Within 15 min, the hydrogen concentration in the heart reaches a plateau concentration of 50 μmol/L. This concentration is higher than the saturated hydrogen concentration achieved by inhalation of 4% hydrogen^19^. In a rat weighing 200 g with usual activity, the respiratory rate is approximately 100 breaths/min, and each breath has a tidal volume of 0.3 mL. At the endpoint, around 1500 mL pure hydrogen gas can be obtained through the respiratory route. However, unlike oxygen, the rapid and efficient utilization of hydrogen in the body is limited due to the absence of abundant hydrogenase and hydrogen storage proteins^20^. The biological utilization rate of oxygen is approximately 20–25%^21^. However, currently, there are no available data regarding the biological utilization rate of hydrogen. Nevertheless, theoretically, it should be much lower than that of oxygen. Additionally, hydrogen is not storable in the body and quickly escapes, so its utilization relies on uninterrupted and prolonged intake. Despite the total hydrogen intake not showing an advantage with magnesium implantation, the localized hydrogen concentration gradient formed by the corroding magnesium slice allowed hydrogen to easily diffuse down its concentration gradient to the surrounding organs^22^. After magnesium slice implantation, the short-term hydrogen concentration in the heart (50 μmol/L) was significantly higher than the concentration of hydrogen in the heart achieved by inhalation (30 μmol/L). Over the next 1, 3, 5, and 7 days, the hydrogen concentration in various organs significantly increased, indicating that this diffusion effect gradually increased with the duration of magnesium slice implantation. These findings also suggest that magnesium slice implantation can maintain continuous and efficient hydrogen release for at least 1 week, covering the entire acute phase of MI^23^.

We also monitored the hydrogen concentration in the gas cavity at different time points. Although not statistically significant, the overall hydrogen concentration showed an increasing trend, possibly due to excessive exchange of generated hydrogen with the surrounding environment caused by the large surface area of the gas cavity. Overall, the benefits to the target organs are undoubtedly dependent on their hydrogen concentration and the duration of hydrogen exposure. We suggest that magnesium implantation for hydrogen production outperforms hydrogen inhalation in terms of the target organ hydrogen concentration that can be achieved and the duration of exposure after reaching that hydrogen concentration.

The safety of magnesium implantation is also an important matter that should be considered. Extensive research has confirmed the favorable safety profile of magnesium, and it is widely applied in the cardiovascular, musculoskeletal^24,25^, and oncology^26^ fields. Moreover, magnesium implantation during the growth stage of children has demonstrated excellent safety^27^. Even in unfavorable conditions, such as renal insufficiency, magnesium implantation does not lead to magnesium ion accumulation^28^. In the present study, blood samples were collected 1 week after MI, and compared to the healthy control (sham) group, there was no evidence of magnesium ion accumulation. All animals showed normal concentrations of sodium, chloride, calcium, and potassium, indicating no electrolyte disturbances. Additionally, markers representing renal excretory function, such as BUN, creatinine, and UA, showed no differences, suggesting that the metabolic response after magnesium implantation is within the body’s tolerable range. Histopathological examination of H&E-stained tissue sections revealed no significant changes in the cellular morphology of the lungs, liver, or spleen, indicating that magnesium implantation did not induce organ toxicity.

As a result of the abundant and sustained hydrogen production provided by magnesium implantation, both histological evaluation of the infarcted myocardium and echocardiography showed that the MI + magnesium group achieved more effective protection against myocardial ischemic injury and had a smaller infarct area. It follows that left ventricular function was better preserved compared with the MI + hydrogen group. Even in patients with complete coronary artery occlusion, hydrogen inhalation effectively increases the hydrogen concentration in the “at-risk” area of MI^6^, and higher hydrogen concentrations in target organs imply better therapeutic effects^25^. It is expected that magnesium implantation, which provides a more sustained and higher concentration of hydrogen to the organs than hydrogen inhalation, would yield superior protective effects. From a mechanistic perspective, in hypoxic cardiomyocytes, the permeability of the mitochondrial inner membrane increases, leading to uncontrolled release of ROS, disrupted redox balance, cell apoptosis, a reduction in the number of cardiomyocytes, and subsequent myocardial remodeling and contractile dysfunction. Hydrogen can ameliorate oxidative damage in MI^29^. Its anti-oxidative effects may be closely related to the protective role of maintaining mitochondrial content and function in cardiomyocytes under oxidative stress^30^. After MI, the mitochondria of cardiomyocytes become damaged, demonstrating swelling, fragmentation, and a fibrous morphology with a decreased MMP. However, both the MI + hydrogen group and the MI + magnesium group exhibited improved mitochondrial and myofibrillar structures, as well as more stable MMPs, with more pronounced improvements observed in the MI + magnesium group due to the higher hydrogen output.

ROS-mediated mitochondrial damage plays an important role in MI^31^. Highly reactive and excessive ROS can quickly overwhelm the endogenous anti-oxidant defense system, triggering the inflammatory response^32^. Oxidative stress in heart failure after MI occurs mainly due to enhanced ROS generation, rather than a decrease in the anti-oxidant defense capacity of the heart^33^. This leads to cell damage through the destruction of lipids, proteins, DNA, and RNA, as well as abnormal mitochondrial DNA replication/transcription and repair following MI^34^. A significant portion of the superoxide formed within the mitochondria cannot pass through the cell membrane, making mitochondrial RNA a vulnerable target for ROS-mediated damage^35^. In contrast, hydrogen rapidly diffuses through the cell membrane and lipid bilayer, reaching the nucleus and mitochondria, where ROS are most likely to be generated^36^. Hydrogen consumption may be closely related to the production of mammalian oxygen radicals^37^. The present study indicated that magnesium implantation had a strong protective effect on mitochondrial RNA. We detected ROS and confirmed the neutralizing effect of hydrogen on excessive ROS. The MI + magnesium group demonstrated a more significant reduction in intracellular ROS than the MI + hydrogen group, which is consistent with the higher hydrogen concentration trend in the MI + magnesium group. Furthermore, magnesium implantation significantly reduced the levels of 8-OHdG and MDA in the infarcted area, indicating that the anti-oxidant effect of magnesium implantation was at least partially guided by ROS clearance. We believe that this beneficial effect was mainly due to the neutralizing properties of hydrogen, which disrupt the detrimental cycle of mitochondrial dysfunction, excessive ROS release, inflammation, and cell damage.

Hydrogen gas has been demonstrated to reduce cell apoptosis^35^. Its anti-apoptotic effects occur in a concentration-dependent manner^38^. Apoptosis is an ongoing process, and the sustained production of hydrogen through magnesium implantation is sufficient to cover the acute phase of apoptosis in MI^39^. The present study examined the changes in apoptotic protein expression (Bax, Bcl-2, cytochrome c, cleaved caspase 9, and cleaved caspase 3) in four groups of rats. The results showed that compared with the sham group (control; no MI), the MI group exhibited upregulation of Bax, cytochrome c, cleaved caspase-9, and cleaved caspase-3 expression, along with downregulation of Bcl-2, indicating enhanced apoptotic signaling. Relative to the MI group, the upregulation of Bax and downregulation of Bcl-2 were reversed to varying degrees in both the MI + hydrogen and MI + magnesium groups, reflecting the ability of hydrogen to protect the mitochondrial integrity, with a stronger effect observed after magnesium implantation, consistent with the mitochondrial electron microscopy results. Upregulation of cytochrome c, cleaved caspase-9, and cleaved caspase-3 expression suggested intrinsic apoptotic pathway activation, which is consistent with previous findings^17^. Compared with the MI + hydrogen group, the MI + magnesium group showed a more significant reduction in the expression of cytochrome c, cleaved caspase-9, and cleaved caspase-3. Overall, the results suggest that compared with hydrogen inhalation, magnesium implantation more effectively inhibits myocardial cell apoptosis by upregulating Bcl-2 and downregulating Bax, cytochrome c, cleaved caspase-3, and cleaved caspase-9 expression, thereby potentially blocking the mitochondrial apoptosis pathway.

## Conclusions

In summary, we used a rat model of MI to evaluate the therapeutic effect of magnesium implantation for hydrogen generation compared with hydrogen inhalation. Magnesium implantation overcomes some of the limitations of hydrogen inhalation in terms of the concentration of hydrogen that can be achieved in the target organs and the duration of exposure. Magnesium implantation allows sustained and stable hydrogen production, allowing target organs to achieve higher hydrogen concentrations than would be possible with hydrogen inhalation. By reducing ROS-mediated mitochondrial damage, oxidative stress, and apoptosis in MI, this approach may offer new insights into the treatment of MI.

### Limitations

This study has some limitations that should be noted. As a result of the impact of air cavity enlargement for more than 1 week on the experimental animals, we only obtained data on the effects of magnesium implantation on MI within 1 week. Although 1 week covered the acute period of MI, we could not obtain magnesium data to examine the effects of magnesium implantation in the subacute and chronic phases of MI. Moreover, microelectrode measurement of organ concentration is greatly affected by the measurement location. Although the measurement location was uniform, the organ anatomy of each animal may have differed; therefore, data fluctuations are inevitable. Furthermore, we implanted 2 g of magnesium slices into rats weighing between 190 and 210 g, equivalent to 1% of their body weight. This experiment represents the initial exploration of hydrogen production from magnesium implantation in the treatment of myocardial infarction. It is important to note the numerous limitations present in human experiments, such as individual tolerance and metabolic differences. When applying the experimental results to human therapy, further research and evaluation on dosage and efficacy must be conducted.

### Supplementary Information

Supplementary Information.

## Data Availability

The datasets used and/or analysed during the current study available from the corresponding author on reasonable request.
