# Supplementary material for: Magnesium implantation as a continuous hydrogen production generator for the treatment of myocardial infarction in rats
Source: Sci Rep. 2024 May 14;14:10959. doi: 10.1038/s41598-024-60609-2 (PMC11094026; doi:10.1038/s41598-024-60609-2)

Bax

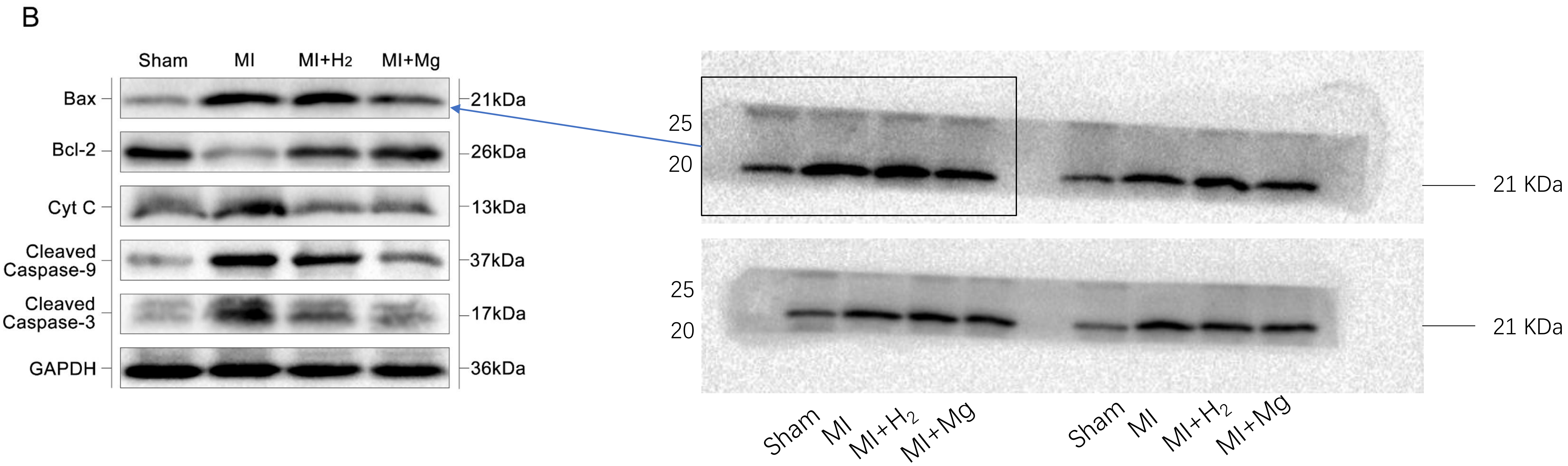

B
Sham
MI
MI+H2
MI+Mg
Bax
Bcl-2
Cyt C
Cleaved Caspase-9
Cleaved Caspase-3
GAPDH
21kDa
26kDa
13kDa
37kDa
17kDa
36kDa
25
20
21 KDa
25
20
21 KDa
Sham
MI
MI+H2
MI+Mg
Sham
MI
MI+H2
MI+Mg

Bcl-2

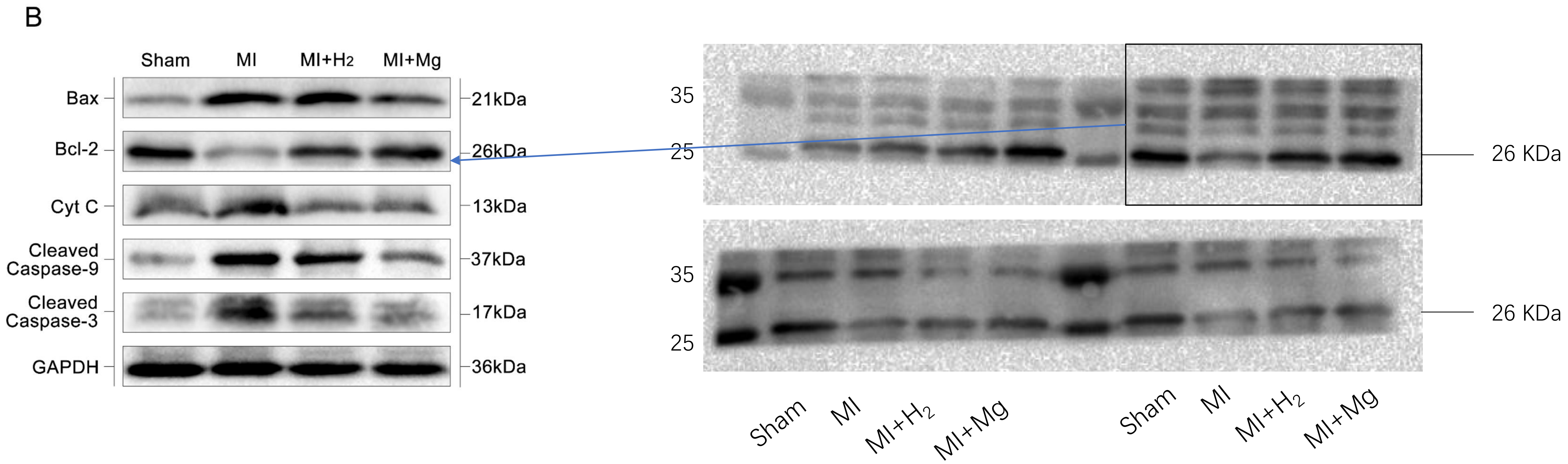

B
Sham
MI
MI+H2
MI+Mg
Bax
Bcl-2
Cyt C
Cleaved Caspase-9
Cleaved Caspase-3
GAPDH
21kDa
26kDa
13kDa
37kDa
17kDa
36kDa
35
25
35
25
26 KDa
26 KDa
Sham
MI
MI+H2
MI+Mg
Sham
MI
MI+H2
MI+Mg

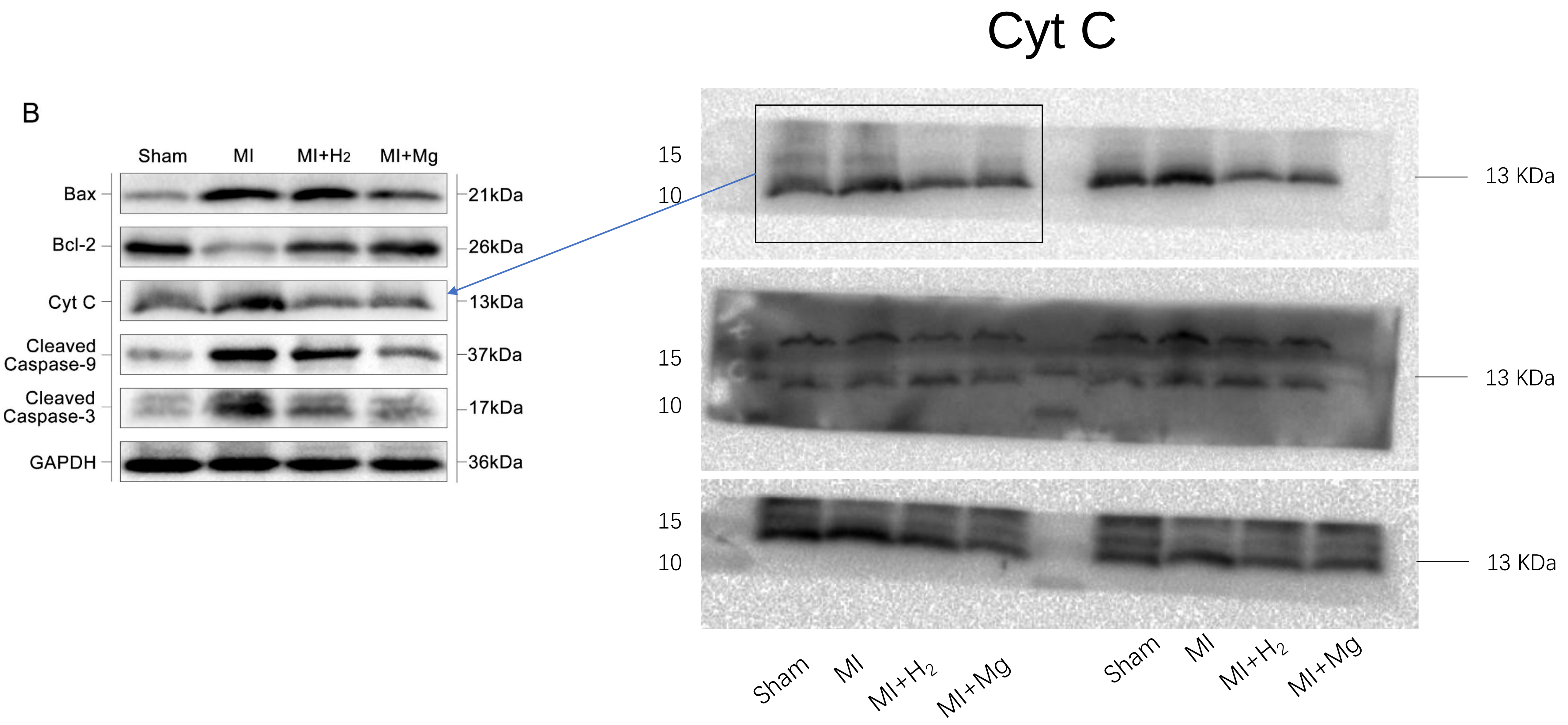
Cyt C
B
Sham
MI
MI+H2
MI+Mg
Bax
Bcl-2
Cyt C
Cleaved Caspase-9
Cleaved Caspase-3
GAPDH
21kDa
26kDa
13kDa
37kDa
17kDa
36kDa
15
10
13 KDa
15
10
13 KDa
15
10
13 KDa
Sham
MI
MI+H2
MI+Mg
Sham
MI
MI+H2
MI+Mg

Cleaved Caspase-9

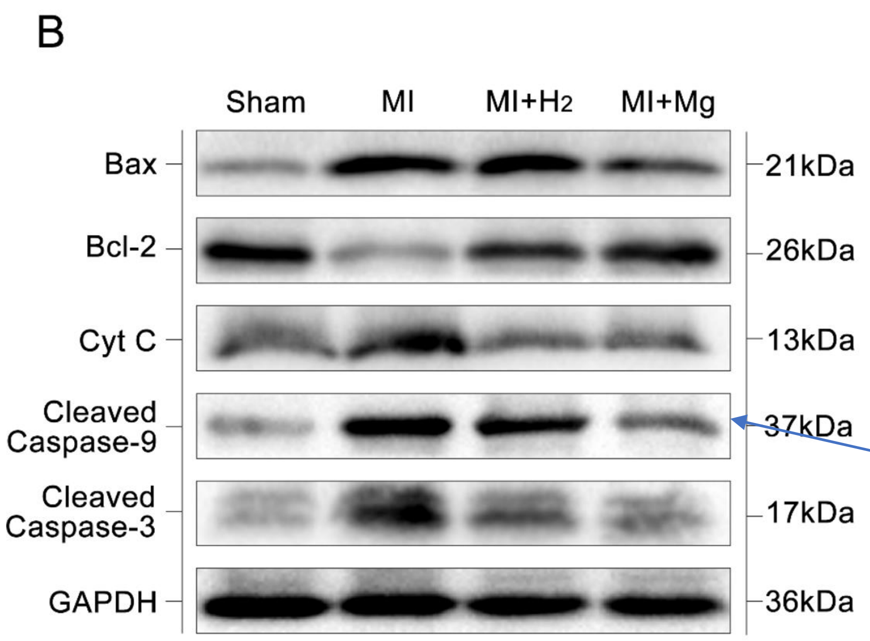

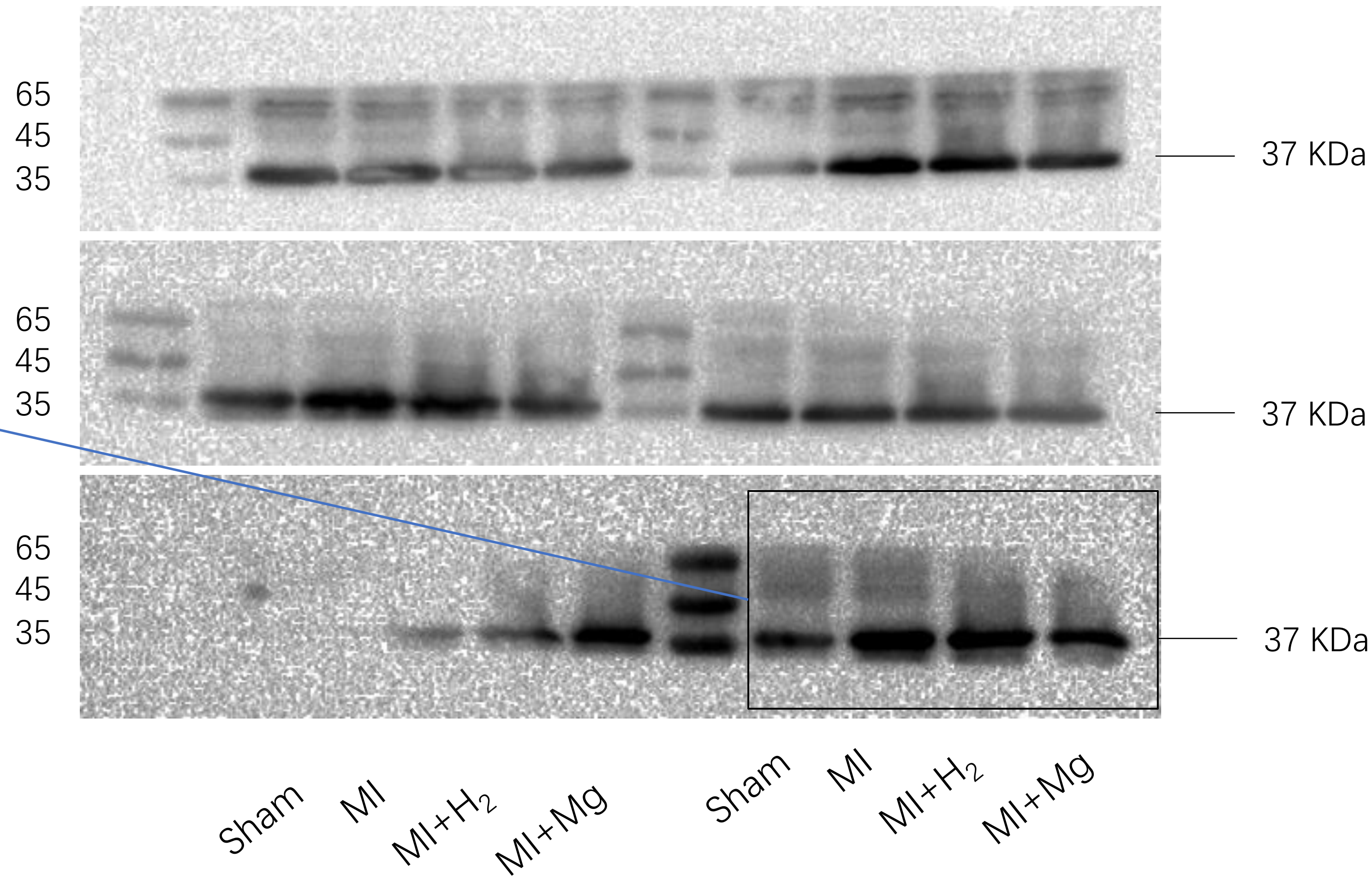

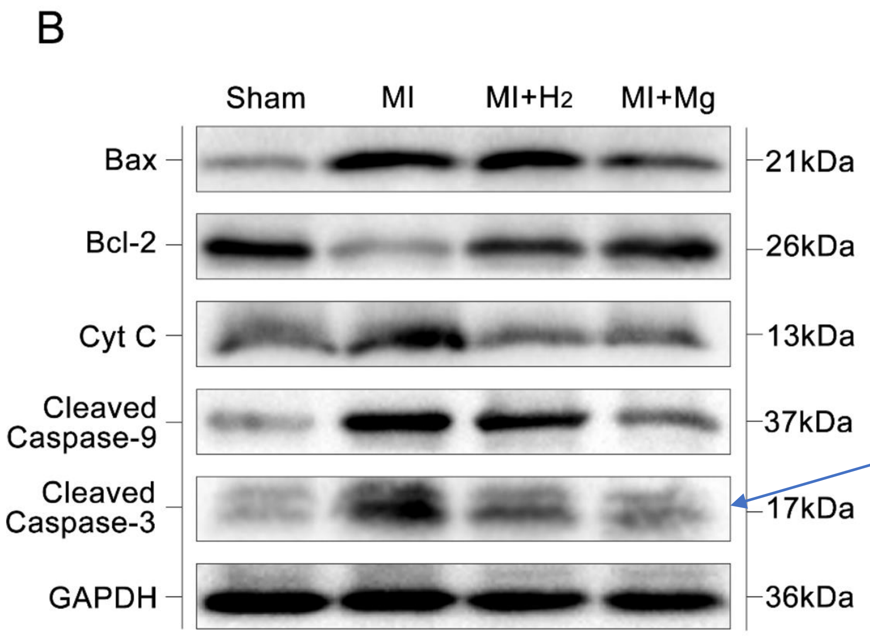
B
Sham
MI
MI+H2
MI+Mg
Bax
Bcl-2
Cyt C
Cleaved Caspase-9
Cleaved Caspase-3
GAPDH
21kDa
26kDa
13kDa
37kDa
17kDa
36kDa

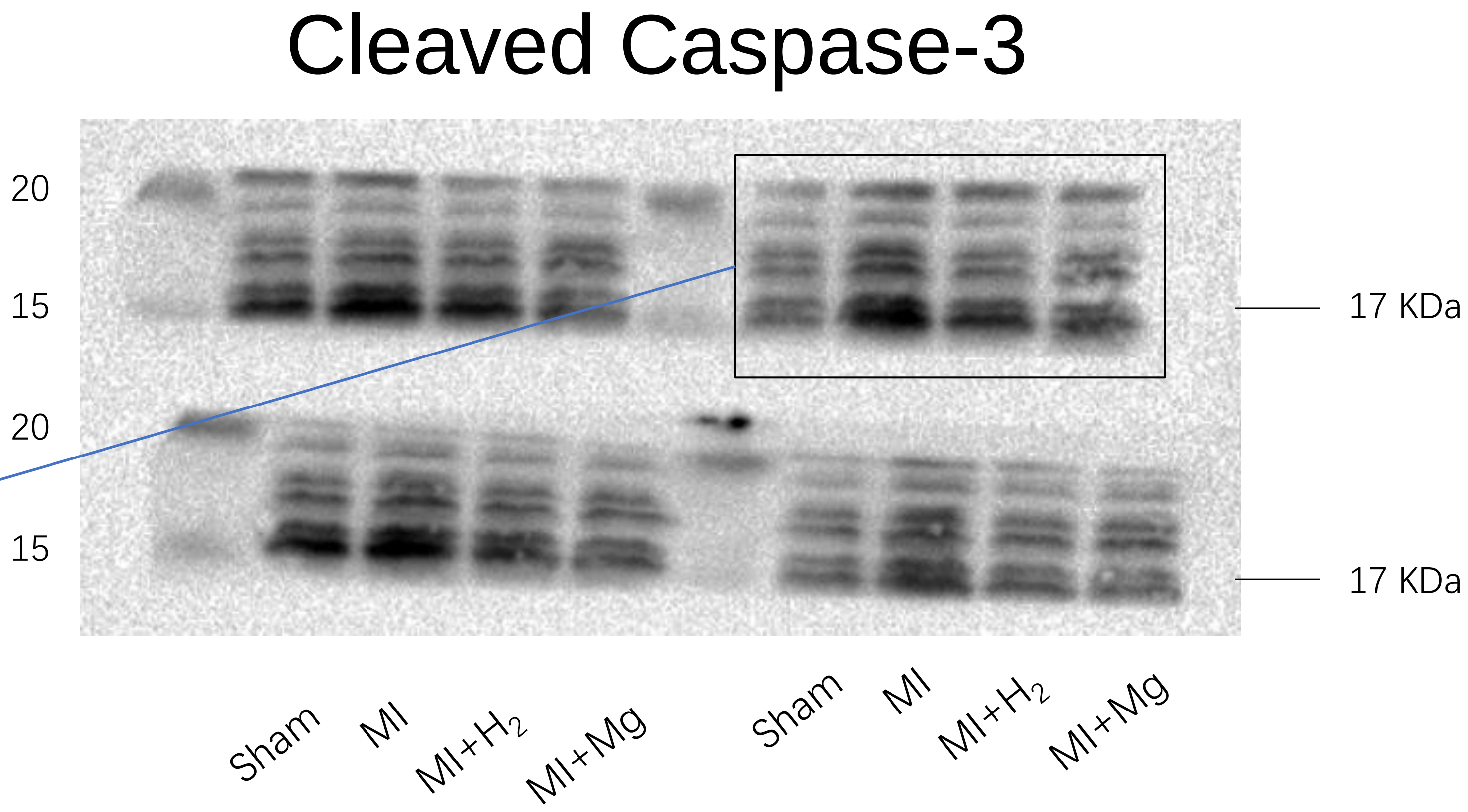
Cleaved Caspase-3
20
15
20
15
17 KDa
17 KDa
Sham
MI
MI+H2
MI+Mg
Sham
MI
MI+H2
MI+Mg

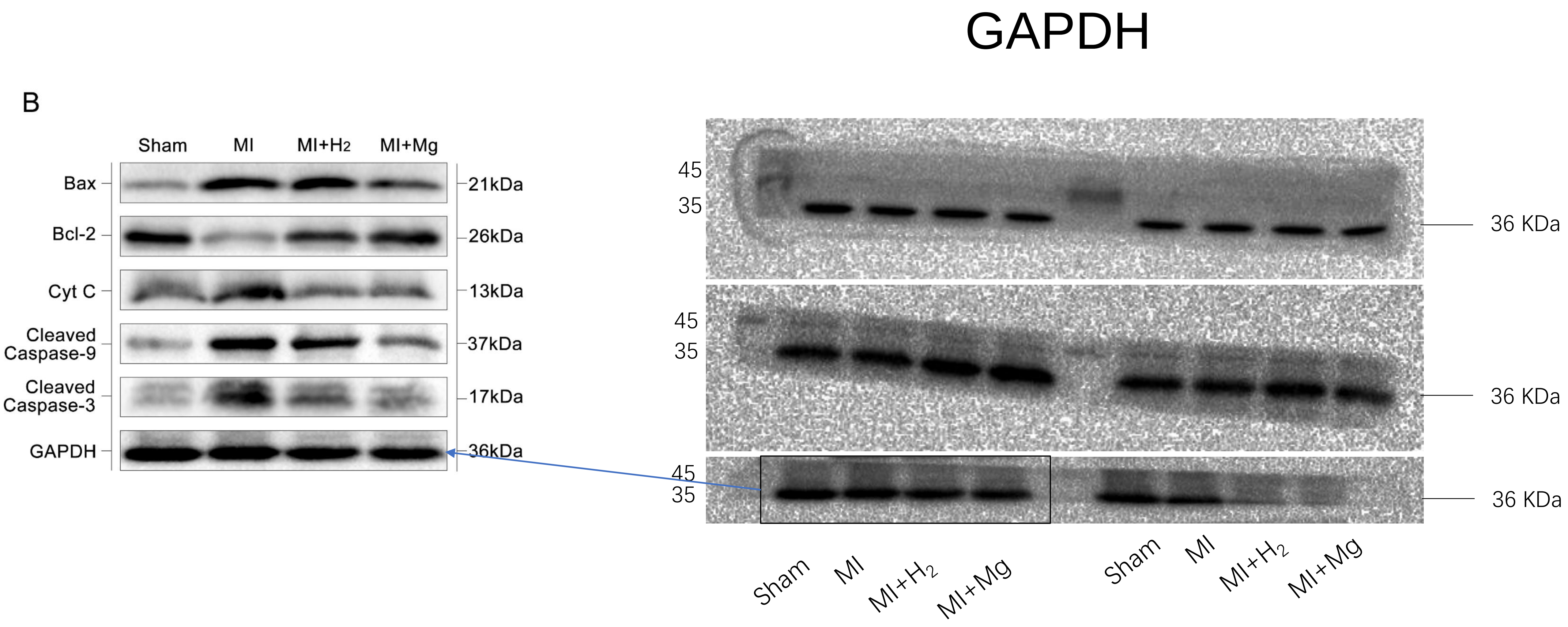
GAPDH
B
Sham
MI
MI+H2
MI+Mg
Bax
Bcl-2
Cyt C
Cleaved Caspase-9
Cleaved Caspase-3
GAPDH
21kDa
26kDa
13kDa
37kDa
17kDa
36kDa
45
35
36 KDa
Sham
MI
MI+H2
MI+Mg
Sham
MI
MI+H2
MI+Mg

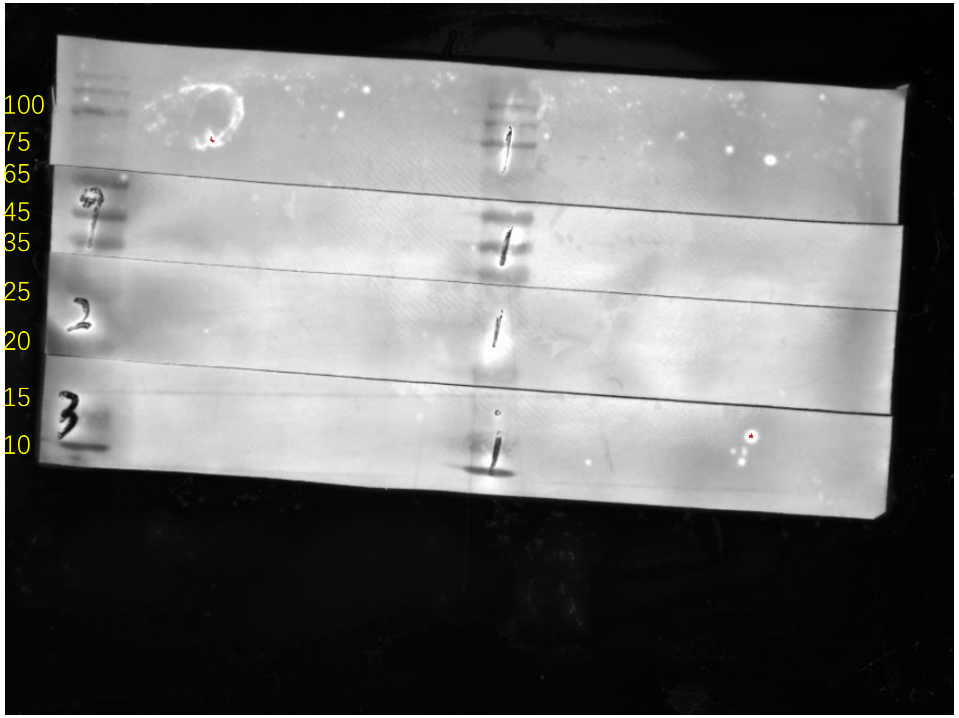

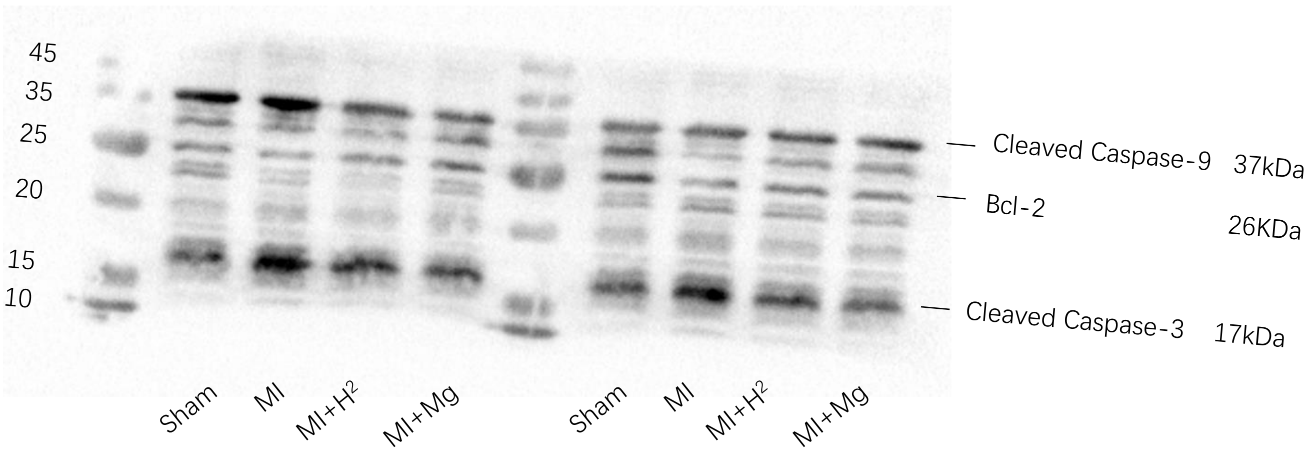

After elution of the antibody,
re-incubation is performed due to the similar molecular weight of the target protein

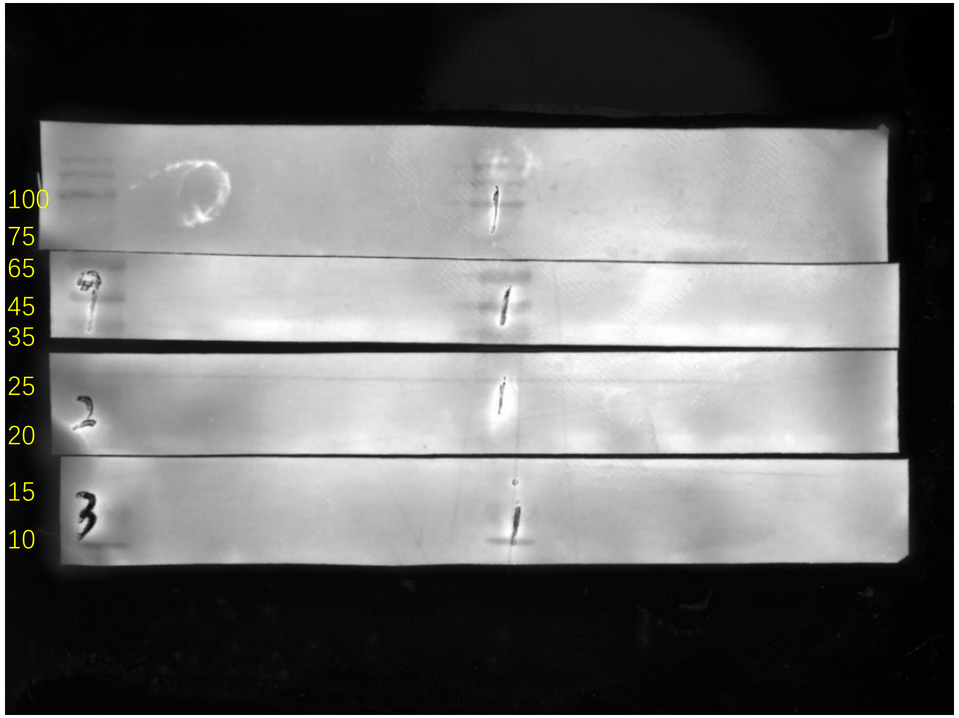

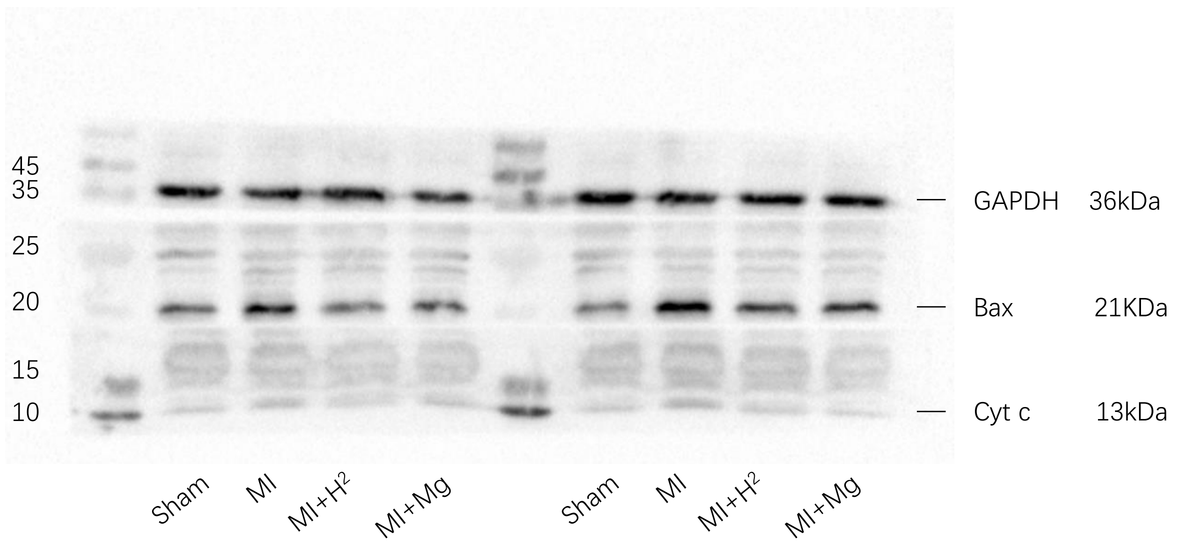

Supplement: Supplementary file 1 — Supplementary Information. [file 41598_2024_60609_MOESM1_ESM.pdf]
